# Analysis of Tracheoesophageal Voice after Total Laryngectomy: A Single Center Experience

**DOI:** 10.3390/jcm13154392

**Published:** 2024-07-27

**Authors:** Andrea Migliorelli, Erennio Natale, Marianna Manuelli, Andrea Ciorba, Chiara Bianchini, Stefano Pelucchi, Francesco Stomeo

**Affiliations:** ENT & Audiology Unit, Department of Neurosciences, University Hospital of Ferrara, via Aldo Moro 8, 44100 Ferrara, Italy

**Keywords:** tracheoesophageal voice, total laryngectomy, voice analysis, voice rehabilitation

## Abstract

**Background/Objectives**: Tracheoesophageal voice is the most commonly used voice rehabilitation technique after a total laryngectomy. The placement of the tracheoesophageal prosthesis can be performed at the same time as the total laryngectomy (primary placement) or in a second procedure after surgery (secondary placement). The purpose of this study is to analyze the substitution voice in patients with a tracheoesophageal prosthesis, considering the influence of radiotherapy and timing of prosthesis placement (primary or secondary) on voice quality. **Methods:** A retrospective analysis was conducted of all patients who received a tracheoesophageal phonatory prosthesis after a total laryngectomy was performed. We assessed whether patients received radiotherapy and whether they had a primary or secondary tracheoesophageal prosthesis. For the voice analysis, maximum phonation time (MPT), INFVo, SECEL, AVQI, CPPS, harmonic to noise ratio (HNR), unvoiced fraction (UVF), and number of voice breaks (NVB) were evaluated. **Results:** A total of 15 patients (14 males and 1 female) with a mean age of 71.8 years (SD ± 7.5) were enrolled. Eight had a primary prosthesis placement and five did not receive radiotherapy. INFVo parameters I and Vo were higher in patients with a primary placement of the phonatory prosthesis (*p* = 0.046 and *p* = 0.047). Patients who received the prosthesis secondarily had a higher mean CPPS and lower mean AVQI. **Conclusions:** A secondary placement of the prostheses seems to result in a minimal advantage in voice quality compared to a primary placement. Radiation therapy, on the other hand, has no effect on voice quality, according to these preliminary data.

## 1. Introduction

Head and neck tumors are the seventh most common cancers in the world. Squamous cell carcinoma is definitely the most common histotype of this tumor type [1].

Laryngeal cancer is one of the most frequent cancers of the head and neck. It is most prevalent in the male population between the ages of 55 and 66. The American Cancer Society estimates 12,380 new cases in 2023 [2].

Despite increasing diagnostic capabilities and awareness in the general population, approximately 60% of new diagnoses to date are of an advanced stage disease (stage III and IV) [3].

According to the National Comprehensive Cancer Network (NCCN) guidelines, a total laryngectomy is a viable treatment option for advanced laryngeal and pharyngolaryngeal cancers and remains the most commonly performed surgery for these patients. In addition, a total laryngectomy is the gold standard for salvage surgery after the failure of radiochemotherapy [4,5,6].

A total laryngectomy includes the definitive separation of the airway and digestive tract with the creation of a permanent tracheostoma. This surgery has a major impact on the patient’s quality of life as it alters the normal functions of swallowing, breathing, and speech and results in a variable degree of an altered sense of smell.

To date, there are basically three methods of speech restoration: esophageal speech, tracheoesophageal speech, and electrolaryngeal speech [7].

In the first two, the speech component is produced at the level of the pharyngolaryngeal region; in the case of electrolaryngeal rehabilitation, an external device placed on the neck produces sounds. In esophageal voice, the patient incubates air at the level of the esophagus, which is subsequently expelled, resulting in a vibration of the pharyngoesophageal segment. Tracheoesophageal voice involves the creation of a tracheoesophageal fistula followed by the placement of a voice prosthesis. The prosthesis allows air to pass from the lungs to the esophagus but prevents the passage of esophageal contents into the trachea [7]. The tracheoesophageal prosthesis was first developed by Blom and Singer in 1980 and it is still the most common rehabilitation option in developed countries [8]. It provides good voice quality and easy patient learning. The main factors that may support the placement of the tracheoesophageal prosthesis are easy mobility of the upper limb, good understanding, and a strong need for vocal communication [9]. Prostheses are still subject to physiological deterioration over time and must be replaced approximately every 4–6 months, or in any case when the patient experiences prosthesis loss. Therefore, one of the main limitations of this technique is the relative proximity of an experienced center to which the patient can be referred for prosthesis replacement when needed [9]. The placement of the tracheoesophageal prosthesis can be performed at the same time as the total laryngectomy (primary placement) or in a second procedure after the surgery (secondary placement) [10]. Although most authors recently prefer primary placement, the increased incidence of perioperative complications associated with concomitant placement is still a source of debate. Several authors have attempted to evaluate the voice of a phonatory prosthesis replacement. However, the literature points to the necessity of standardized measurement tools for the acoustic evaluation of substitution voices [7]. In addition, there are no studies comparing the substitution voice in relation to the timing of a phonatory prosthesis placement.

Our hypothesis is that tracheoesophageal voice quality may be influenced by the timing of tracheoesophageal prosthesis insertion and radiation therapy. Therefore, the aim of this study is to analyze the substitution voice in patients with a tracheoesophageal prosthesis using acoustic analysis, perceptual assessment, and self-assessment. Additionally, the influence of radiation therapy and timing of prosthesis insertion (primary vs. secondary) on the voice was evaluated.

## 2. Materials and Methods

This is a retrospective study. All patients who received a tracheoesophageal phonatory prosthesis after a total laryngectomy from 2016 to 2022 at the ENT Department of the Sant’Anna University Hospital of Ferrara were evaluated.

The inclusion criteria were:

(i) Placement of the tracheoesophageal phonatory valve for at least six months.
(ii) The phonatory valve was used daily by the patient.
(iii) Tracheoesophageal voice was the main mode of verbal communication.
(iv) Voice assessment by acoustic analysis.

Exclusion criteria were: (i) laryngectomized patients using other alternative voices (e.g., esophageal voice or laryngophone); (ii) patients not using the tracheoesophageal voice daily; (iii) voice acoustic analysis was not available. Furthermore, patients surgically treated before 2016 were not assessed in terms of acoustic voice analysis; thus, in order to analyze uniform records, they were excluded.

The following information was retrieved for each patient: age, sex, date of laryngectomy, time of tracheoesophageal prosthesis placement (primary: placed at the same time as the laryngectomy; secondary: placed in a second surgery distant from the laryngectomy), time between prosthesis placement and activation (in days), and number of rehabilitation sessions.

Voice recordings were made with a Samson MeteorMic condenser microphone (Samson Technologies, Hauppauge, NY, USA) connected via USB to a notebook (IdeaPad 5, Lenovo, Lenovo Group Limited, Beijing, China) at a distance of approximately 20 cm from the subject under standard conditions, i.e., in a quiet environment (ambient noise < 40 dB) and with constant recording volume. The recordings were made using Audacity^®^ recording software version 2.1.2 (Audacity Team, GNU General Public License), with the project sampling rate set to 44.100 Hz; recordings were saved in 16-bit digital format.

At the beginning of the recording, the amplification level was adjusted to the phonation intensity of each patient and then kept constant throughout the recordings in order to obtain recordings with approximately the same amplitude for all patients.

There were three types of obtained recordings:

− Three sustained vowels (-a-) for as long as possible. This recording was used to measure the maximum phonatory time (MPT) and to calculate the Acoustic Voice Quality Index (AVQI) (by cutting a 3 s mid-vowel fragment).
− Five sentences from the Italian version of the CAPE-V (Consensus Auditory Perceptual Evaluation-V), the first two of which were then used for the AVQI calculation [11].
− The patient’s attempt to perform a glissando, i.e., a smooth increase and subsequent decrease in the frequency of the sound produced between two extreme tones, achieved by placing the tonal pitches between the two tones themselves in continuity. This phenomenon, which in normal phonation is produced by the tension and relaxation of the vocal cords, is more limited in patients with phonatory prostheses and requires the “training” of the mucous membrane of the neoglottis, which does not naturally have this ability to stretch.

Patients were asked to produce the neo-voice at a comfortable pitch and intensity.

The present study was conducted in accordance with the Declaration of Helsinki (2008). It was conducted retrospectively through a systematic review of hospital case histories and therefore did not affect patient care in any way, as it was limited to the creation of a database and its evaluation.

### 2.1. Voice Assessment

#### 2.1.1. Perceptual Assessment—INFVo

For the perceptual assessment of voice, the INFVo scale was used [12].

This scale consists of the following parameters.

I: Overall impression. This parameter analyzes the overall quality of the voice; it indicates the combined impression caused by all the characteristics of the voice.

N: Unwanted additive noise. This parameter analyzes the level of annoyance caused by the audibility of all types of uncontrolled noise produced during speech.

F: Fluency. This parameter analyzes the perceived smoothness and fluidity of sound production and depends significantly on the patient’s ability to manage exhaled air.

Vo: Voice quality. This parameter analyzes the voice produced and assesses whether it is vocal or non-vocal; voices that produce a lot of breath noise and contain few or no vocal segments score low.

Each parameter was scored from 0 (very good substitution voice) to 10 (very deviant substitution voice) via a visual scale by a group of voice professionals consisting of two physicians specializing in otolaryngology and phoniatrics (AM and EN). The recordings were re-evaluated in a quiet environment (ambient noise < 40 dB).

#### 2.1.2. Acoustic Analysis and AVQI Scores

Editing and analysis of the recordings were performed using Praat software (Version 6.0.37 for Mac, Boersma and Weenink, University of Amsterdam, Amsterdam, Netherlands). In particular, the 3 s mid-vowel (SV, sustained vowel) and the 25 syllables of the first two sentences of the CAPE-V (CS, continuous speech) were used; the AVQI score was calculated through the script “ACOUSTIC VOICE QUALITY INDEX (AVQI) v.03.01” validated for the Italian language, obtaining the combined scores for each patient [11].

Additionally, through the same script, the ‘combined’ cepstral peak prominence smoothed (CPPS), i.e., derived from both sustained “a” and continuous speech, was extracted.

Finally, through the PRAAT voice report, other acoustic parameters were obtained, such as MPT, Harmonic to Noise Ratio (HNR), Unvoiced Fraction (UVF), Number of Voice Breaks (NVB), and the minimum and maximum frequencies of the glissando attempt.

### 2.2. Self-Assessment Tool—Secel Questionnaire

Patients were given the I-SECEL [Self-Evaluation of Communication Experiences after Laryngeal Cancer—Italian version] questionnaire [13], which measures communication experiences after laryngeal cancer through self-evaluation.

It is an instrument that detects the subject’s adaptation to substitution voice through 35 questions divided into three categories: general, environmental, and attitudinal.

The questionnaire is designed so that the score for a patient who is well adapted to the tracheo-esophageal voice should be 36, with a standard deviation of 12.

Patients with a score higher than 60 are considered to be poorly adapted to the new mode of communication and specific counseling is recommended to improve their communication experience.

### 2.3. Statistical Analysis

Descriptive analysis was performed on all variables with their frequencies. Numerical data were expressed as absolute values, percentages, mean ± standard deviation, or median (range).

For numerical variables analyzed, the Kolmogorov–Smirnov test was used to test the normality of the distribution.

Continuous numerical data, expressed as mean and standard deviation, were analyzed using Student’s *t*-test as a parametric test. For ordinal categorical data, the Mann–Whitney U test was used.

All *p*-values less than 0.05 were considered statistically significant. All analyses were performed with the Statistical Package for the Social Sciences (SPSS) 29.0.1 software (IBM Corp., Armonk, NY, USA).

## 3. Results

Of the 30 patients who underwent tracheoesophageal prosthesis placement during the analyzed period, only 15 met the inclusion criteria and were therefore included in the study. Fourteen of these were males and only one female was included.

The mean age of the patients at the time of voice recording was 71.8 years (SD ± 7.5; range 62–83) with a mean follow-up time after the total laryngectomy of 4.3 years (SD ± 3.1).

Eight patients had a primary tracheoesophageal fistula and the other seven had a secondary fistula; in the latter group, the mean latency between the laryngectomy and prosthesis placement was 43.3 months (SD ± 28.1). Postoperative radiotherapy was performed in 66.7% of the patients.

Table 1 shows the demographic characteristics of the sample analyzed.

All patients included in the study are users of the Provox Vega permanent voice prosthesis (Atos Medical AB, Hörby, Sweden). Further details on the characteristics of the prosthesis used can be found in Table 2. Table 3 summarizes all the results of the voice analysis.

Radiation therapy had no effect on the voice quality of the phonatory prosthesis, with none of the results obtained being statistically significant (Table 4). None of the SECEL domains are statistically significant.

However, comparing primary and secondary prosthesis outcomes, the former appear to have a longer time between apposition and activation than the latter (*p* = 0.005).

In contrast, no significant differences were found between the two groups in the number of speech therapy sessions, MPT, HNR, and UVF.

Patients who received the prosthesis secondarily had a higher mean CPPS and a lower mean AVQI; this difference does not reach statistical significance but still has a trend towards significance (Table 5).

On the other hand, the analysis of INFVo shows that the parameters I and Vo are higher in patients with a primary placement of the phonatory prosthesis (*p* = 0.046 and *p* = 0.047), while N has a trend towards significance without reaching it. The difference in the F parameter is not statistically significant. There are no statistically significant differences between the two groups in the SECEL self-assessment.

## 4. Discussion

According to the results of the present study, the secondary placement of the prostheses seems to result in a minimal advantage in voice quality compared to the primary placement. Radiation therapy, on the other hand, has no effect on voice quality, according to these preliminary data.

Laryngeal cancer causes about 80,000 deaths per year and is the most common head and neck cancer in Italy [14,15]. The population studied was mainly male, with a mean age of 71.8 years (±7.5 years); this sample reflects the findings in the literature [9]. Total laryngectomy is indicated for advanced tumors or salvage surgery, with excellent results for survival and disease-free time. However, complete removal of the larynx results in significant changes in breathing, swallowing, speech, taste, and smell. In addition, a definitive tracheostomy has a negative impact on the patient’s quality of life [16].

The ability to speak is a fundamental human function that distinguishes us from animals. Therefore, much research has been done over the years to try to restore speech after a total laryngectomy.

Nowadays, there are three possible voice rehabilitation techniques after a total laryngectomy: esophageal voice, tracheoesophageal voice, and electrolaryngeal voice [7,17].

Since the 1980s, the voice prosthesis has become the standard of care for voice rehabilitation after a total laryngectomy, with success rates ranging from 65% to 85% for primary placement and 69% to 83% for secondary placement [18].

The tracheoesophageal prosthesis can be placed during surgery (primary) or after surgery (secondary). Secondary placement usually occurs at least 6 months after the completion of treatment [19].

The advantage of a primary placement is that patients undergo a single operation and can begin speech rehabilitation early. However, this choice is associated with an increased risk of surgical complications (such as fistula, puncture site leakage, stoma stenosis, and local infection). In addition, patients with secondary prostheses may have more reasonable expectations regarding voice quality and thus be more satisfied with their voice after the postoperative voiceless period [20,21].

Therefore, the aim of this study is to analyze the substitution voice in patients with a tracheoesophageal prosthesis, considering the influence of radiotherapy and the timing of prosthesis placement (primary or secondary) on voice quality. A retrospective analysis of the voice of 15 patients who received a tracheoesophageal phonatory prosthesis after a total laryngectomy was performed using acoustic and aerodynamic analysis, perceptual evaluation, and self-assessment.

Concerning the timing of prosthesis placement, the results within our small sample seem to show a minimal advantage in terms of voice quality for patients who underwent secondary placement compared to primary, especially in terms of INFVo scores. No significant difference was found for the acoustic and aerodynamic parameters and for the SECEL questionnaire. Radiation therapy has no effect on voice quality according to these preliminary data. Despite the small sample size, our results seem to be consistent with the existing literature.

In addition, the present study shows that patients with a secondary placement have a shorter activation time than those with a primary placement. Of course, total laryngectomy surgery can result in complications that can delay the activation of the prosthesis itself.

In a systematic review of 37 articles, Barauna Neto et al. [10] found an increased risk of infection in patients with primary prosthesis placements. In addition, periprosthetic leaks were found in 10% fewer patients with secondary placements than with primary placements.

In 2018, a systematic review by Van Sluis et al. [7] compared the three main methods of vocal rehabilitation after surgery. The authors conclude that tracheoesophageal voice shows superior results in acoustic parameters, fundamental frequency, MPT, and intensity compared to the other methods.

Some authors use the Voice Handicap Inventory (VHI 30) for subjective voice assessment [7,9]. This test, developed in 1997, is used to assess the severity of voice handicap and would be intended for dysphonic patients; that is, patients with the presence of the larynx [22].

To date, most studies evaluating substitution voices have used the MPT, INFVo, and SECEL as parings.

The mean MPT in our case series is 7.2 (±3.2). This result is consistent with what has been found in the literature [9]. This result is not influenced by the timing of prosthesis placement.

The INFVo is a scale for perceptual evaluation and rating of prosthetic devices. It was introduced in 2006 by Moerman et al., and it evaluates five parameters: impression, intelligibility, noise, fluency, and vocality [23]. Parameter I assess the overall voice quality and intelligibility; N the annoyance caused by uncontrolled noise during speech; F fluency; and Vo whether the voicing should be vocal or non-vocal. This rating scale appears to be more appropriate than the GRBAS for evaluating substitution voices. In the present case series, the parameters I and Vo were higher in patients with a primary affixation of a phonatory prosthesis. Therefore, using this scale, patients with a secondary prosthesis seem to have a better voice than the other group.

The SECEL scale, on the other hand, quantifies the patient’s level of adaptation to the substitution voice. It analyzes three domains (general, environmental, and social) that affect oral communication. Usually, the environmental domain is the most impaired [24].

The mean value we found for the total SECEL score is 31.2 (±11); a similar result (28.1) was also found by Mesolella et al. in 55 patients [25]. In the present series, no differences were found between those who received the prosthesis as a primary or secondary implant. In our study, we also used the CPPS and AVQI to perform an acoustic analysis of the voice.

Substitution voice is often a phenomenon characterized by the massive presence of aperiodicity and noise, due to the peculiar way in which it is generated, namely by the vibration of a mucosa that is not specifically structured for the phonatory function. In most cases, it is not possible to identify a fundamental frequency in this vibration, which is why it seems inappropriate to use those parameters (i.e., perturbation parameters such as jitter) that are based precisely on the identification of the F0.

Currently, there is no unanimous consensus on the acoustic analysis of the substitution voice, and the topic is debated. We believe that these two analyses, especially the CPPS (which does not necessarily require strict identification of a fundamental frequency), are important for objective acoustic evaluation of substitution voices and can be incorporated into routine clinical practice for voice evaluation in patients with phonatory prostheses [26,27].

We found that patients who wear the prosthesis secondarily have a higher mean CPPS and a lower mean AVQI and therefore a better voice. These results do not reach full statistical significance and should be confirmed in further studies.

To the best of our knowledge, there are no studies in the literature that have evaluated substitution voice with these analyses and therefore we cannot compare our results.

Instead, when comparing the voice of those who received radiotherapy and those who did not, we found that the only difference reaching a borderline statistical significance (*p* = 0.057) was in the G-domain of the SECEL. This parameter was higher in those who did not receive radiotherapy.

In recent years, scientific work has increasingly focused on possible treatment options with organ preservation and similar oncologic outcomes [6,28,29,30].

Therefore, partial laryngectomies can be performed in the early stages of the disease and in selected advanced cases.

In 2014, Succo et al. [28] classified open partial horizontal laryngectomies (OPHLs) into three groups: supraglottic I, supracricoid II, and supratracheal III.

A partial laryngectomy offers numerous advantages to the patient, including closure of the tracheostoma and the ability to speak due to preservation of the cricoarytenoid unit [31].

Studies analyzing the voice of patients after an OPHL have emerged with the increasing number of publications on this surgery. Patients who have undergone a type I OPHL have a better voice than other types of OPHLs. This result can be attributed to the preservation of the vocal cords in these patients (in contrast to OPHL II and III) and the removal of the supraglottic region exclusively [31].

In 2022, Fantini et al. [31] found an MPT of 9.1 s for patients who underwent an OPHL type II and 6 s for those who underwent an OPHL type III.

The same article also shows that the average total SECEL score is 34.8 and 40.4 for patients with an OPHL type II and III, respectively.

If we compare the authors’ results with our case history, we find that patients who underwent a total laryngectomy followed by vocal rehabilitation with phonatory prosthesis have worse MPT scores than OPHL II but better than OPHL III.

On the other hand, analyzing the SECEL, we find that both OPHLs considered have worse values than phonatory prostheses.

Finally, the overall INFVo is lower in an OPHL than in rehabilitation with phonatory prostheses.

Similar results were found by D‘Alatri et al. [32]. The authors compared the voices obtained with an OPHL with those obtained with phonatory prostheses and found that the vocal parameters I and Vo were significantly higher in the TL group.

In conclusion, based on our small sample and the current literature, it seems that rehabilitation with a phonatory prosthesis after a total laryngectomy is comparable to an OPHL type II and III from the point of view of voice quality. Notably, based on previous studies, we could preliminarily hypothesize that there is no significant difference between the voice obtained with a tracheoesophageal prosthesis and the voice of a type II OPHL; on the other hand, the tracheoesophageal voice seems to have a better voice outcome than a type III OPHL. Obviously, we know that these assumptions are severely limited by the difficult comparability of samples across studies, as well as by the complexity of the issue, which requires further investigation.

It is also important to highlight that the choice of surgery to be performed (partial vs. total laryngectomy) should be based primarily on oncologic radicality. It should also be emphasized that OPHLs are usually more accepted by the patients, since the presence of the definitive tracheostoma has a strong negative impact on the patient’s quality of life and sociality.

Major drawbacks of this study are: (i) the small number of patients included, (ii) representing the experience of a single institution, (iii) the retrospective nature of the study, and (iv) the presence of potential confounders (i.e., sex was not a balanced dataset among the studied group).

## 5. Conclusions

Nowadays, a total laryngectomy is still the most common surgical procedure for the treatment of laryngeal cancer. Tracheoesophageal voice is the most commonly used voice rehabilitation technique.

To the best of our knowledge, the literature lacks standardized protocols for the analysis of substitution voice. In our study, we propose some parameters that we believe could be essential for substitution voice analysis.

In addition, the MPT, SECEL, and INFVo are included in all recent studies analyzing substitution voices and therefore should be routinely used to evaluate them.

The secondary placement of prostheses seems to result in a minimal advantage in voice quality compared to primary placements. Radiation therapy, on the other hand, has no effect on voice quality.

Finally, we pointed out that tracheoesophageal voice seems to have similar overall results to a type II OPHL and slightly better than a type III OPHL.

Further prospective and multicenter studies with larger case series are necessary to confirm the results of this study.

## Figures and Tables

**Table 1:** Demographics and patients‘ characteristics.

	n	%
Gender		
Female	1	6.7%
Male	14	93.3%
Mean Age (years) [±SD]	71.8 [±7.5]	
Smoking Habit		
Yes	15	100%
No	0	0%
Staging		
pT		
pT2	3	20%
pT3	6	40%
pT4a	6	40%
pN		
pN0	5	33.3%
pN1	6	40%
pN2a	1	6.7%
pN2c	1	6.7%
pN3b	2	13.3%
RT		
Yes	8	66.6%
No	5	33.4%
Mean FUPT after TL (years) [±SD]	4.3 [±3.1]	
Timing prothesis insert		
Primary	8	53.3%
Secondary	7	46.7%

Abbreviation legend. RT: Radiotherapy; SD: Standard Deviation; TL: Total Laryngectomy; FUPT: Follow-Up Time.

**Table 2:** Prothesis characteristics.

Size of Prothesis	Number of Patients
6 mm, 22.5 F	8
8 mm, 20 F	1
8 mm, 22.5 F	5
10 mm, 22.5 F	1

**Table 3:** Voice Analysis.

Patient	RT	MPT	HNR	UVF	NVB	CPPS	AVQI	F Gliss. Min–Max	I	N	F	Vo	INFVo TOT	G	E	A	GEA TOT
1	Y	7.62	2.163	98.02	0	4.98	7.55	75.8–498.5	4	5	5	4	18	15	5	0	20
2	Y	7.17	3.417	49.65	6	7.35	7.25		6	7	6	6	25	11	20	15	46
3	Y	5.57	0.059	59.56	7	6.92	6.74	74.4–409.7	2	2	1	1	6	13	10	2	25
4	N	2.29	2.659	58.41	7	4.12	9.68		7	8	9	8	32	10	28	15	53
5	Y	11.72	6.391	33.73	3	7.28	6.79		9	9	7	9	34	11	10	7	28
6	N	8.54	2.106	92.6	2	4.32	9.1	272.9–442.1	6	7	8	7	28	9	12	9	30
7	Y	5.22	3.568	44.21	13	4.03	8.49	75.4–329.7	4	6	7	6	23	9	15	10	34
8	Y	6.27	1.202	55.61	22	6.53	8.14	77.8–460.1	5	6	7	6	24	12	13	3	28
9	N	2.13	2.005	62.38	5	6.39	7.19	73.7–332.5	3	4	4	2	13	9	9	4	22
10	Y	9.07	2.827	95.79	0	4.18	9.17	337.3–421.3	3	6	4	3	16	13	22	9	44
11	N	11.76	8.471	22.08	10	12.05	3.68	75.1–185.6	5	5	7	6	23	4	10	4	18
12	Y	11.94	2.422	59.278	11	6.54	7.43	65.0–488.7	2	4	1	2	9	15	2	1	18
13	N	5.22	3.383	42.775	14	9.75	5.12	74.1–394.1	3	5	2	4	14	13	11	1	25
14	N	8.99	2.776	13.408	23	5.65	8.09	78.0–333.6	5	6	4	5	20	7	17	8	32
15	Y	3.85	5.686	7.087	2	8.58	6.17	75.4–173.2	2	5	7	3	17	8	18	19	45
Mean (±SD)		7.2 (±3.2)	3.3 (±2.1)	53 (±27.8)	8.3 (±7.2)	6.6 (±2.3)	7.4 (±1.6)		4.4 (±2)	5.7 (±1.7)	5.3 (±2.5)	4.8 (±2.3)	20.1 (±7.9)	10.6 (±3)	13.5 (±6.7)	7.1 (±5.8)	31.2 (±11.1)

Legend. SD: Standard Deviation; Y: Yes; N: No; RT: Radiotherapy; MPT: Maximum Phonatory Time; HNR: Harmonic to Noise Ratio; UVF: Unvoiced Fraction; NVB: Number of Voice Breaks; CPPS: cepstral peak prominence smoothed; AVQI: Acoustic Voice Quality Index; I: Overall impression; N: Unwanted additive noise; F: Fluency; Vo: Voice quality; G: General; E: Environmental; A: Attitudinal.

**Table 4:** Patients Receiving Radiotherapy vs. Patients Not Receiving Radiotherapy.

	RT (±SD)	NoRT (±SD)	*p*-Value
Time PtA	12.1 (10.7)	8.6 (10.5)	0.287
NoRS	5 (3.3)	2.8 (2.2)	0.097
MPT	7.6 (2.8)	6.5 (3.9)	0.282
HNR	3.1 (2)	3.6 (2.5)	0.348
UVF	55.9 (28.4)	48.6 (28.9)	0.320
CPPS	6.3 (1.5)	7 (3.2)	0.267
AVQI	7.5 (0.9)	7.1 (2.3)	0.331
NVB	7.1 (7.2)	10.2 (7.5)	0.334
I	4.1 (2.3)	4.9 (1.6)	0.310
N	5.6 (1.9)	5.9 (1.5)	0.857
F	5 (2.5)	5.7 (1.5)	0.547
Vo	4.4 (2.5)	5.3 (2.2)	0.438
G	11.9 (2.4)	8.7 (3)	0.057
E	12.7 (6.7)	14.5 (7.2)	0.906
A	7.3 (6.6)	6.8 (4.9)	0.953
SECELToT	31 (10.8)	30 (12.3)	0.723

Legend. SD: Standard Deviation; Time PtA: time between prosthesis placement and activation (in days); NoRS: number of rehabilitation sessions; MPT: Maximum Phonatory Time; HNR: Harmonic to Noise Ratio; UVF: Unvoiced Fraction; NVB: Number of Voice Breaks; CPPS: cepstral peak prominence smoothed; AVQI: Acoustic Voice Quality Index; I: Overall impression; N: Unwanted additive noise; F: Fluency; Vo: Voice quality; G: General; E: Environmental; A: Attitudinal.

**Table 5:** Primary vs. Secondary Prosthesis Placement Comparison.

	Primary (±SD)	Secondary (±SD)	*p*-Value
Time PtA	17.3 (±10)	3.2 (2.5)	0.005
NoRS	4.9 (3.7)	3.4 (1.9)	0.198
MPT	6.8 (2.7)	7.5657 (3.9)	0.336
HNR	2.6 (1.9)	3.9 (2.3)	0.141
UVF	61.4 (22.5)	43.2 (31.7)	0.116
CPPS	5.7 (1.5)	7.6 (2.7)	0.065
AVQI	7.9 (1.1)	6.7 (1.9)	0.072
NVB	7.5 (7)	9.3 (7.7)	0.643
I	5.4 (2)	3.3 (1.3)	0.046
N	6.3 (2.1)	5 (0.8)	0.067
F	6.2 (2.4)	4.1 (2.3)	0.086
Vo	5.8 (2.5)	3.6 (1.5)	0.047
G	11.3 (2)	9.8(3.9)	0.448
E	14.1 (7.1)	12.7 (6.8)	0.728
A	7.6 (5.8)	6.6 (6.3)	0.684
SECELToT	33 (11.1)	29 (11.5)	0.324

Abbreviation legend. Time PtA: time between prosthesis placement and activation (in days); NoRS: number of rehabilitation sessions; MPT: Maximum Phonatory Time; HNR: Harmonic to Noise Ratio; UVF: Unvoiced Fraction; NVB: Number of Voice Breaks; AVQI: Acoustic Voice Quality Index; SD: Standard Deviation.

## Data Availability

Data are contained within the article.
